# Development, organization, and remodeling of phoronid muscles from embryo to metamorphosis (Lophotrochozoa: Phoronida)

**DOI:** 10.1186/1471-213X-13-14

**Published:** 2013-04-24

**Authors:** Elena N Temereva, Eugeni B Tsitrin

**Affiliations:** 1 Department of Invertebrate Zoology, Biological faculty, Moscow State University, Moscow 119992, Russia; 2 Institute of Developmental Biology, Russian Academy of Sciences, Moscow 117808, Russia; 3 Department of Invertebrate Zoology, Biological Faculty, Lomonosov State University, Leninskie Gory 1/12, Moscow 119992, Russian Federation

## Abstract

**Background:**

The phoronid larva, which is called the actinotrocha, is one of the most remarkable planktotrophic larval types among marine invertebrates. Actinotrochs live in plankton for relatively long periods and undergo catastrophic metamorphosis, in which some parts of the larval body are consumed by the juvenile. The development and organization of the muscular system has never been described in detail for actinotrochs and for other stages in the phoronid life cycle.

**Results:**

In *Phoronopsis harmeri*, muscular elements of the preoral lobe and the collar originate in the mid-gastrula stage from mesodermal cells, which have immigrated from the anterior wall of the archenteron. Muscles of the trunk originate from posterior mesoderm together with the trunk coelom. The organization of the muscular system in phoronid larvae of different species is very complex and consists of 14 groups of muscles. The telotroch constrictor, which holds the telotroch in the larval body during metamorphosis, is described for the first time. This unusual muscle is formed by apical myofilaments of the epidermal cells. Most larval muscles are formed by cells with cross-striated organization of myofibrils. During metamorphosis, most elements of the larval muscular system degenerate, but some of them remain and are integrated into the juvenile musculature.

**Conclusion:**

Early steps of phoronid myogenesis reflect the peculiarities of the actinotroch larva: the muscle of the preoral lobe is the first muscle to appear, and it is important for food capture. The larval muscular system is organized in differently in different phoronid larvae, but always exhibits a complexity that probably results from the long pelagic life, planktotrophy, and catastrophic metamorphosis. Degeneration of the larval muscular system during phoronid metamorphosis occurs in two ways, i.e., by complete or by incomplete destruction of larval muscular elements. The organization and remodeling of the muscular system in phoronids exhibits the combination of protostome-like and deuterostome-like features. This combination, which has also been found in the organization of some other systems in phoronids, can be regarded as an important characteristic and one that probably reflects the basal position of phoronids within the Lophotrochozoa.

## Background

The Phoronid a is a phylum of the Bilateria, but the position of phoronids among other bilaterians is still unclear. Although recent molecular data indicate a close relationship between phoronids and typical Spiralia [1,2] or even as “in-group brachiopods” [3], this relationship is not yet supported by comparative anatomy and embryology. Although new data on protostomian features in phoronid embryology have recently been published [4], these results do not allow the definite establishment of phoronids as typical protostomian animals [5]. The obtaining of new and detailed information about phoronid development will help answer questions about the phoronid phylogeny. The study of myogenesis in particular can contribute to phylogenetic analysis because an understanding of myogenesis can clarify some questions of bilaterian phylogeny and reveal homology between some muscle elements in different bilaterian taxa (for details see [6,7]). In polyplacophorans, for example, the arrangement of muscles is metameric after metamorphosis but is mesh-like before metamorphosis [6,7]. This suggests the absence of metamery in the common bilaterian ancestor [6,7].

Phoronid myogenesis has never been studied in detail. Although the mesoderm formation has been traced with light microscopy [8] and with modern methods of experimental biology [9,10], the fine structure and location of muscle cells in the early embryo have not been described. All phoronid species have a planktotrophic larva called the actinotrocha, which lives in plankton for 1 to 3 months [11]. Among phoronids, only *Phoronis ovalis* has a lecithotrophic creeping larva, which does not have tentacles and which undergoes metamorphosis in seven days. According to some data obtained with light and laser confocal microscopy [8,12-14], the organization of the muscular system in planktotrophic phoronid larvae is very complex. At the same time, the use of transmission and scanning electron microscopy established the location and organization of known muscles in phoronid larvae and also indicated the existence of new muscular elements that may play a main role in larval behavior and metamorphosis [15].

Adult phoronids have a unique body plan that forms during catastrophic metamorphosis. The general view is that during the metamorphosis of phoronid larvae into adults, most larval systems including the muscular system are lost and definitive organs form *de novo*[12]. This view, however, was not consistent with our observations [15,16]. These studies point out several differences in the ontogenetic patterns of muscle development, but the potential plasticity of these patterns including the overall larval myoanatomy of phoronid larvae remains largely unknown. Therefore, the current study investigates myogenesis, the organization of the larval muscular system and its fate during the metamorphosis of *Phoronopsis harmeri*.

## Results

### Myogenesis

**Figure 1:**
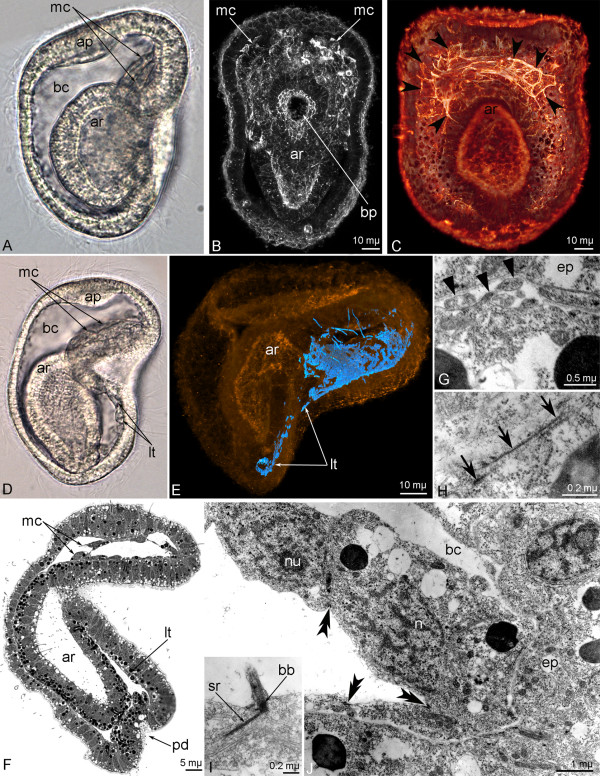
**First steps of *Phoronopsis harmeri *myogenesis.** In all images, the apical is to the top, and the ventral is to the right, except in B and C where the ventral side is toward the reader. **A**. Photograph of live mid-gastrula. **B**. Confocal image of the midgastrula stained with phalloidin; z-projection of 20 middle optical sections; frontal view. **C**. Mid-gastrula stained with phalloidin, 3D-projection of ventral side viewed from the dorsal side. The muscular cells (arrowheads) spread on the ventral wall of the embryo, above the archenteron (ar). **D**. Late gastrula with myoepithelial cells (mc), which have immigrated into the posterior part of the embryo and formed two lateral tiers (lt). **E**. Late gastrula stained with phalloidin, 3D-projection of first muscular system (blue) in the embryo (yellow); lateral view. **F**. Sagittal semi-thin section of late gastrula, in which hindgut begins to form as a posterior depression (pd) of the ectoderm. **G** – **J**. Ultrastructural organization of the first muscle cells, which have immigrated from the archenteron anterior wall; sagittal sections of the embryo. **G**. Basal processes (closed arrowheads) of muscle cells. **H**. First myofilaments (arrows) in the basal cytoplasm of muscle cells. **I**. The cilium with basal body (bb) and striated rootlet (sr) in the muscle cell. **J**. Part of the lining of the anterior pole of the late gastrula. The lining is formed by myoepithelial cells, which connect to each other via desmosomes (double arrowheads) and have a nucleus (n) with a nucleolus (nu). Abbreviations: ap – apical plate; bc – blastocoels; bp – blastopore; ep – epidermis.

Muscle cells differentiate from mesodermal cells, which have immigrated from the anterior ectodermal–endodermal boundary (the anterior wall of the archenteron) in the mid-gastrula stage (Figure 1A). Mesodermal cells have a strong actin-myosin net under the cell membrane and can be recognized with phalloidin labeling (Figure 1B). Mesodermal cells occupy the blastocoels above the blastopore opening (Figure 1C). In the late gastrula, mesodermal cells surround the blastopore and pass to the posterior part of the embryo by two tiers (Figure 1D,F). This passing results from the activity of individual cells and simultaneous cell proliferation. In the anterior part of the late gastrula, mesodermal cells form a continuous layer in which all cells are connected by desmosomes (Figure 1E,J). All cells, which are immigrated from the archenteron, bear a short cilium with a basal complex consisting of a basal body, an accessory centriole, and a short, striated rootlet (Figure 1I). These cilia are probably obtained from the archenteron epithelial cells, which already have cilia. All mesodermal cells are flat, and a round nucleus containing a nucleolus occupies the center of each cell (Figure 1J). Lateral parts of the cell form long, wide processes, which spread on the basal lamina (Figure 1J). The basal border also forms some processes (Figure 1G). At the late gastrula stage, the first bundles of myofilaments appear in the basal cytoplasm of some cells (Figure 1H). These cells form the muscular system. In the late gastrula, the muscular system consists of the continuous layer of the cells above the blastopore.

**Figure 2:**
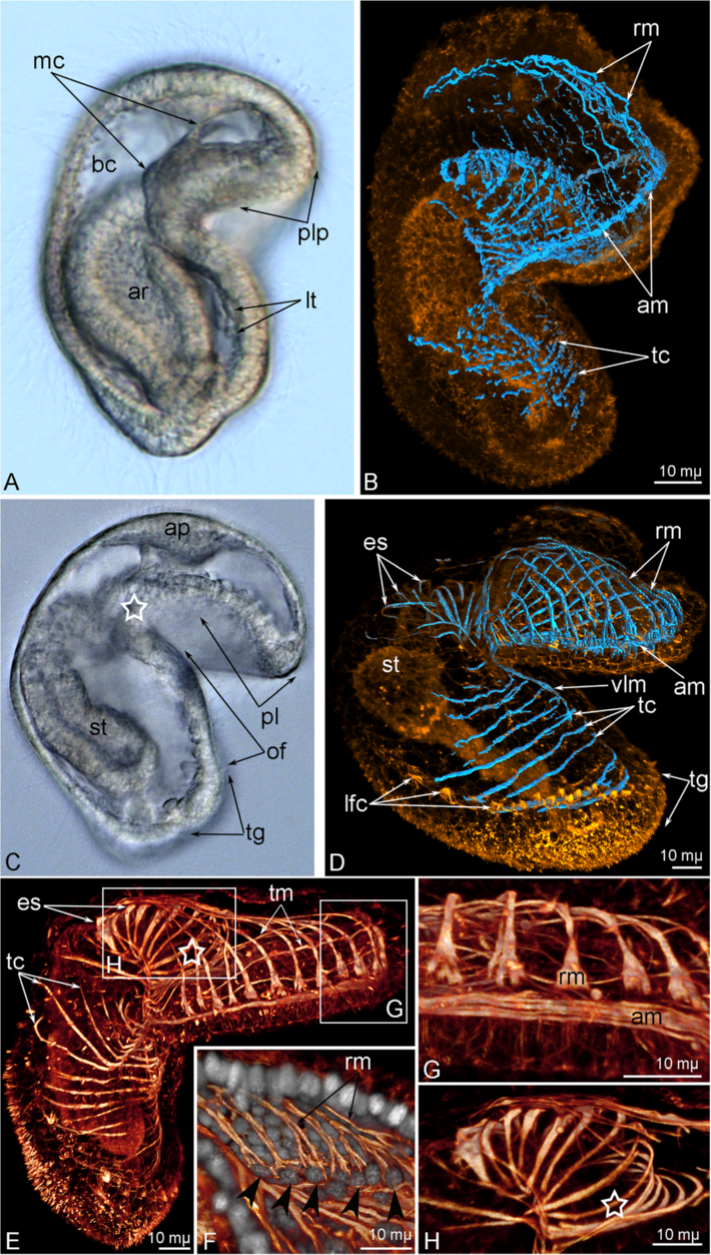
**Organization of the muscular system in early and young larva of *Phoronopsis harmeri*.** In all photographs, animals are viewed from the lateral side; the ventral side is to the right, and apical side is to the top. **A**, **C**. Photographs of live larvae. **B**, **D**. 3D-reconstructions of muscular system (blue) of larvae stained with phalloidin (yellow). **A**, **B**. Early actinotrocha. **C**, **D**. Young larva. **E** – **H**. 3D-reconstructions of muscular system of a young larva stained with phalloidin (**E** – **H**) and with Hoechst (**F**). The position of the mouth is indicated by asterisk. **E**. Whole larva. **F**. Internal surface of the preoral lobe with radial muscles (rm) and their basal nuclei (arrowheads). **G**. Musculature of the edge of the preoral lobe includes annular muscle (am) and the bases of radial muscles. **H**. Esophageal musculature. Abbreviations: ap – apical plate; ar – archenteron; bc – blastocoels; es – esophageal musculature; lfc – laterofrontal cells are marked by long thick microvilli; t – lateral tiers of muscle cells; mc – muscle cells; pl – preoral lobe; plp – precursor of the preoral lobe; of – oral field; st – stomach; tc – transverse collar muscles; tg – tentacular ridge; tm – transversal muscles of the preoral lobe; vlm – ventro-lateral muscle.

In the early actinotrocha, the precursor of the preoral lobe (= the hood) forms (Figure 2A). The latter arises as a growth of the ventral part of the embryo anterior pole. The precursor of the preoral lobe consists of two walls: an upper wall, which bears the apical plate, and a lower wall, which lies in front of the mouth (blastopore opening). In the early actinotrocha, the continuous muscular layer in the preoral lobe precursor separates into singular wide fibers (Figure 2B). They form semicircles around the upper wall of the esophagus. Muscle fibers are concentrated along the edge of the small preoral lobe and form the future *annular muscle of the hood* (Figure 2B). The number of the muscle fibers on the upper wall of the preoral lobe increases. Muscle cells form radial processes, which extend from the annular muscle to the apical plate. Under the mouth, thin muscle fibers are located transversely on the ventral body wall (Figure 2B).

In the young actinotrocha, which has a complete intestine and begins to feed, the muscular system is stronger than in earlier stages. The body of the young larva is divided into a large preoral lobe and a collar, which has a spacious oral field on the ventral side (Figure 2C). The collar is the body part between the preoral lobe and the tentacular ridge, which bears latero-frontal cilia and extends along the lateral and ventral sides of the collar region (Figure 2C). At this stage, the preoral lobe of the larva has a well-developed muscular system (Figure 2D, E). It consists of one annular muscle, 20–22 thick radial muscles, and nine thin transverse muscles. The annular muscle is composed of sveral circular muscle fibers (Figure 2G); it forms two short branches, which continue from the preoral lobe to the ventrolateral sides of the oral field (Figure 2D). Radial muscles extend along the upper wall of the preoral lobe, whereas the transverse muscles lie on the lower wall of the preoral lobe. In each radial muscle, the distal portion of the cell, which contacts the annular muscle, is wide and contains a large nucleus (Figure 2F,G). Here, myofilamets are grouped in two, three, or four bundles (Figure 2F). The proximal portion of the radial muscle is thin and ends on the dorsal side of the preoral lobe (Figure 2E). The 3–4 most distal transverse muscles, which are situated near the edge of the preoral lobe, start and end in the annular muscle, whereas the proximal muscles that are located near the mouth form circles. Most of the proximal transverse muscles form the circular musculature of the mouth and the esophagus (Figure 2H). In the transverse muscle cells, the nucleus mainly occupies the central part. The muscular system of the collar region is represented by 8–10 transverse muscles, which are strong on the ventral side and weak on the lateral sides. These *transverse muscles of the collar* are located repetitively from the mouth to the tentacular ridge (Figure 2D).

**Figure 3:**
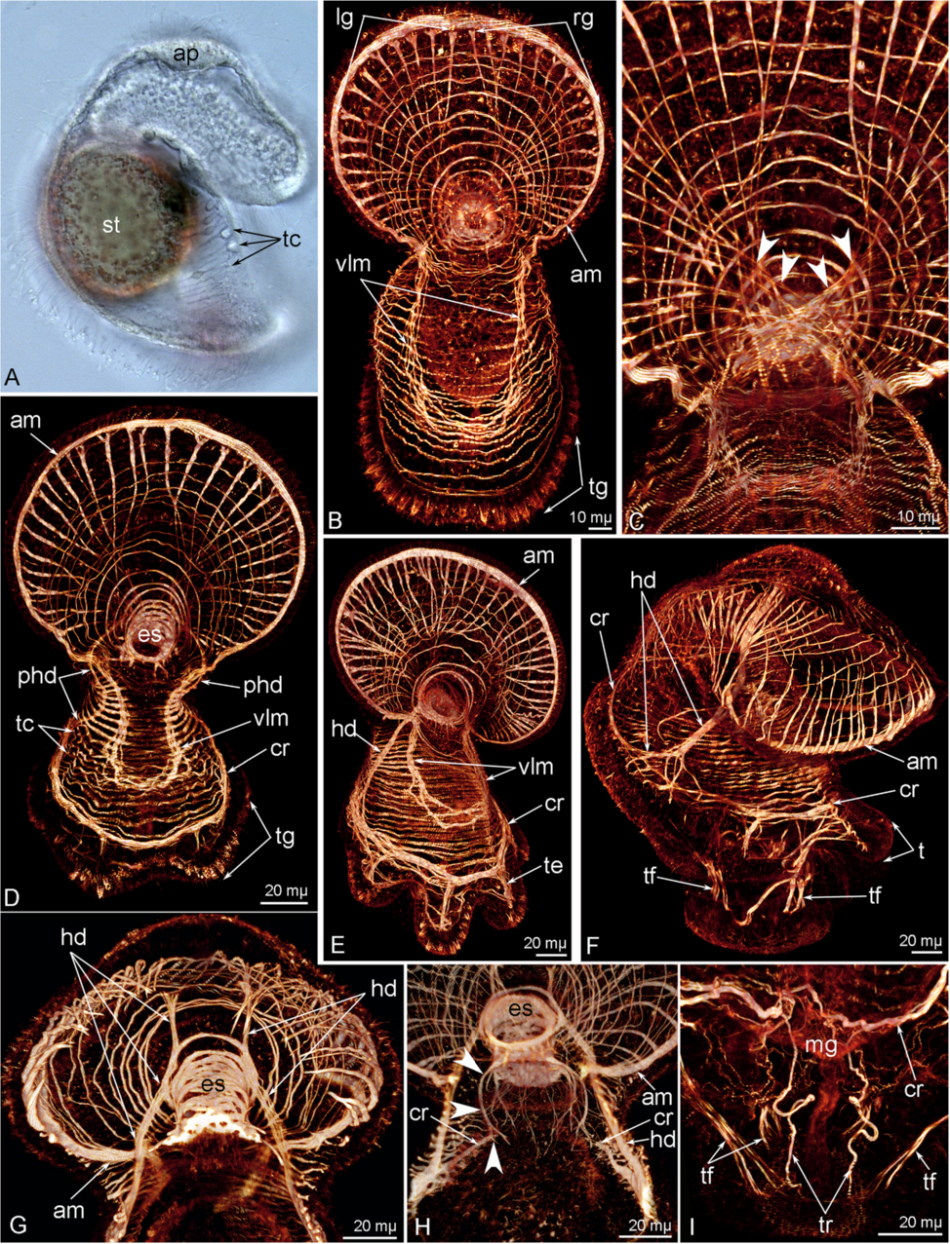
**Organization of the muscular system in different larval stages of *Phoronopsis harmeri*.** In all photographs, the apical pole of larva is to the top. **A**. Live 6-day-old larva, lateral view, ventral side is to the right. **B** – **I**. 3D-reconstructions of the muscular system of larvae stained with phalloidin. **B**. Whole 6-day-old larva viewed from the ventral side. **C**. Dorsal side of the base of the preoral lobe in 6-day-old larva. The chiasm of the radial muscles is indicated by arrowheads. **D**. A 9-day-old larva viewed from the ventral side. **E**. An 11-day-old larva viewed from the ventrolateral side. **F**. A 15-day-old larva, lateral view, ventral side is to the right. **G**. Anterior body part of a 15-day-old larva viewed from the dorsal side. The most dorsal optical sections have been excised. **H**. Anterior part of a 15-day-old larva viewed from the ventral side. The most ventral optical sections have been excised. Processes of the esophageal muscle cells, which contact the collar ring muscle, are shown by arrowheads. **I**. The trunk of a 24-day-old larva, ventral view. Abbreviations: am – annular muscle of the preoral lobe; cr – collar ring muscle; es – esophageal musculature; hd – hood depressors; lg – left group of radial muscles of the preoral lobe; mg – midgut; phd – precursor of the hood depressor; rg – right group of radial muscles of the preoral lobe; t – tentacle; tc – transverse collar muscles; te – tentacle elevator; tf – telotroch flexor; tg – tentacular ridge; tr – trunk retractor; vlm – ventrolateral muscles.

In the 6-day-old larva, the muscular system becomes more complex (Figures 3A-C). The ventro-lateral branches of the annular muscle become longer, pass to the tentacular ridge, and merge on the ventral side of the oral field (Figure 3B). The number of radial muscles of the preoral lobe increases to 34. Radial muscles are separated into rather similar left and right groups (Figure 3B). The thin proximal ends of the muscles of the left group join together, extend to the right side of the preoral lobe, and attach to the right point of the preoral lobe base (Figure 3C). The proximal ends of the muscles of the right group intertwine unite, extend to the left side of the preoral lobe, and attach to the left point of the preoral lobe base. Thus, on the dorsal side of the preoral lobe, the radial muscles form a chiasm (Figure 3C). The esophagus is surrounded by 12–14 circular muscles. The number of the transverse muscles of the collar increases to 15–17 (Figure 3B).

In the 9-day-old larva, one pair of primordial tentacles develops (Figure 3D). The *collar ring muscle* appears as a result of fusion of several transverse muscles of the collar. Short radial muscles extend from the collar ring muscle into each tentacle (Figure 3D). With age, the number of tentacles increases; in the 11-day-old larva, tentacle elevators originate in each tentacle and pass from the base to the terminal end of the tentacle (Figure 3E).

In the 9-day-old larva, the *hood depressor* precursors appear. These paired muscles pass from the esophagus along the lateral side of the collar region to the collar ring muscle (Figure 3D). In later stages, the hood depressors become more evident.

In the 15-day-old larva, each hood depressor forms several long branches near the collar ring muscle (Figure 3F). The anterior end of each depressor grows from the esophagus to the basal lamina of the apical plate (Figure 3G). The hood depressors connect the apical pole of the larva and the collar ring muscle. When passing near the esophagus, the hood depressors contact the strong esophageal muscular system, which consists of several thick circular muscles (Figure 3H). The esophageal muscle cells form processes, which pass posteriorly and contact to the dorsal parts of the collar ring muscle (Figure 3H). In the 15-day-old larva, the trunk forms as a short body part under the tentacle ridge, and its muscular system begins to become evident (Figure 3F). Two groups of muscles appear simultaneously: the *telotroch flexors* and the *trunk retractors* (Figure 3I). The telotroch flexors are represented by two pairs of muscles, which pass from the telotroch base to the collar ring muscle. Trunk retractors are two curled, thin muscles attached to the epidermis near the anus and to the collar ring muscle (Figure 3I).

### Organization of the muscular system in advanced larvae

**Figure 4:**
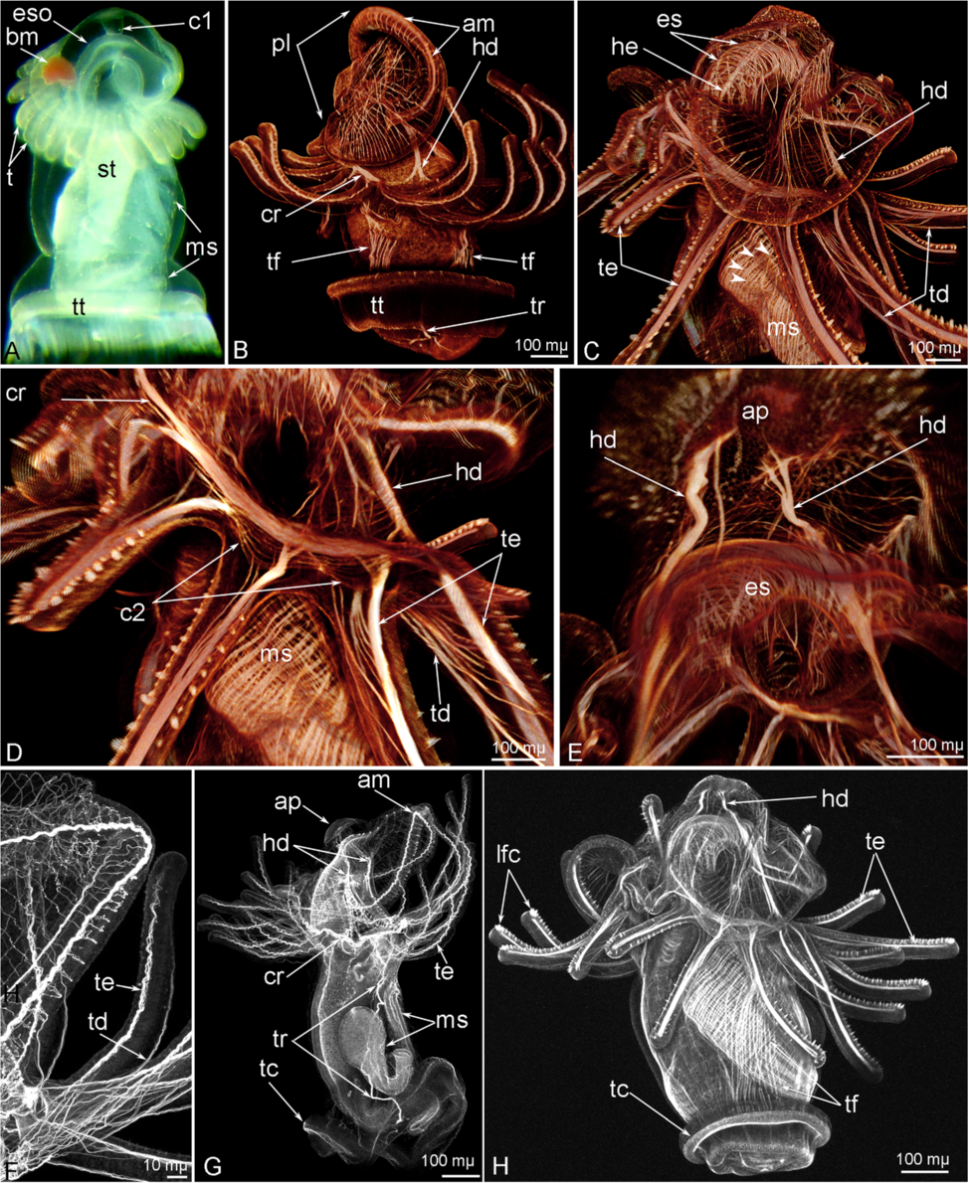
**Organization of muscular system in advanced and competent larvae of *Phoronopsis harmeri.*** In all photographs, the apical pole of the larva is to the top. **A**. Photograph of live larva, lateral view, the ventral side is to the right. **B** – **E**. 3D-reconstructions of the muscular system of larvae stained with phalloidin. **F – H.** Z-projections of the muscular system of larvae stained with phalloidin. **A**. Competent larva. **B**. Overview of the musculature of a competent larva; lateral view. **C**. Musculature of anterior part of an advanced larva, lateral view. **D**. Collar region of an advanced larva with tentacular coelom (c2), collar ring muscle (cr), tentacle elevators (te), and tentacle depressors (td); lateral view, the ventral side is to the right. **E**. Apical body part of an advanced larva with apical plate (ap), paired hood depressors (hd), and esophageal musculature (es); dorsal view. **F**. Tentacular musculature of a competent larva. **G**. Overview of the musculature of a competent larva; lateral view, the ventral side is to the right. All main muscles are shown. **H**. Overview of the musculature of an advanced larva; lateral view, the ventral side is to the right. Abbreviations: bm – blood mass; es – esophageal musculature; eso – esophagus, lfc – laterofrontal cells of postoral ciliated band; ms – metasomal sack; st – stomach; t – tentacle; tt – telotroch.

Advanced and competent phoronid larvae have a very complex muscular system, which is composed of the following elements: the annular muscle of the hood, radial and circular (transverse) muscles of the hood, hood depressors (one pair), hood elevators (= hood retractor) (one pair), esophageal musculature, transverse muscles of collar, the collar ring muscle, tentacle elevators, tentacle depressors, trunk retractors (one pair), telotroch flexors (two pairs), trunk body musculature including muscles of the blood vessels, the telotroch constrictor, metasomal sack musculature (Figure 4).

Some of these muscles (hood depressors, trunk retractors, and telotroch constrictor) play important roles in future metamorphosis. The function of these muscles can be clearly observed in live larvae undergoing metamorphosis (Additional file 1).

**Figure 5:**
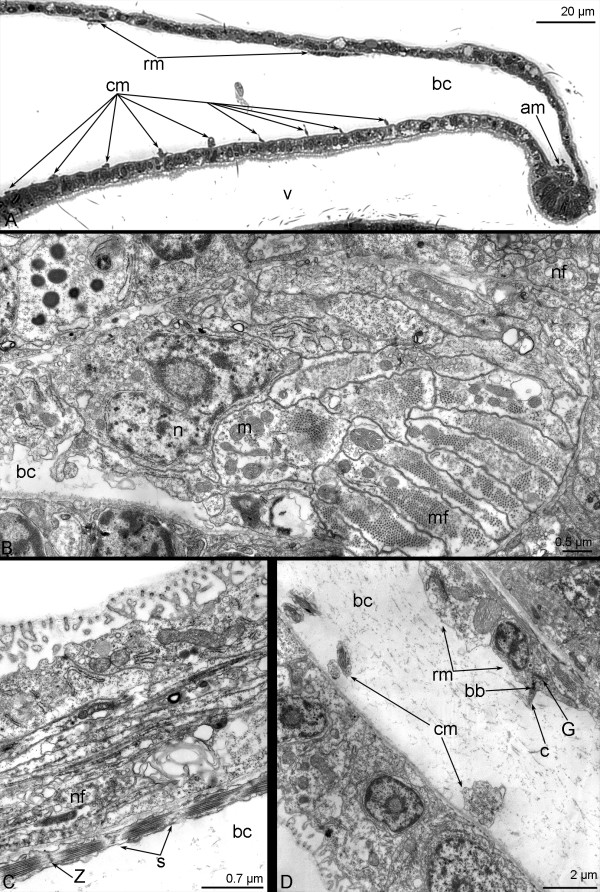
**Organization of the hood musculature in *Phoronopsis harmeri *larvae.** longitudinal semithin (**A**) and thin (**B**-**D**) sections. A. Anterior part of the hood. **B**. Annular muscle. **C**. The projection of the radial muscle cell. **D**. Organization of the muscle cell. Abbreviations: am – annular muscle; bc – blastocoel; cm – circular muscle; m – mitochondrion; mf – myofilaments; n – nucleus; nf – nerve fibers; rm – radial muscle; s – sarcomere; v – vestibulum; Z – Z-line. bb – basal body; c – cilium; G – Golgi apparatus.

In advanced larvae, the annular muscle extends along the edge of the preoral lobe (Figures 5A, B). The annular muscle consists of 12–15 muscle cells, whose projections contain myofilaments and mitochondria (Figure 5B). In the preoral lobe, the radial muscle cells pass along the upper wall, whereas the circular muscle cells are located on the lower wall (Figure 5A). Circular muscles are distant from the edge of the preoral lobe, are regularly arranged in the middle of the hood, and increase in density towards the esophagus. In muscle cells, the myofilaments form sarcomeres, which are divided by Z-lines that are represented by electron-dense bodies (Figure 5C). The cross-striated organization of the muscle cells is evident even in semithin sections (Figure 5A). Muscle cells are monociliar; the basal body, the second centriole, and Golgi apparatus are located in the cilium basis (Figure 5D).

**Figure 6:**
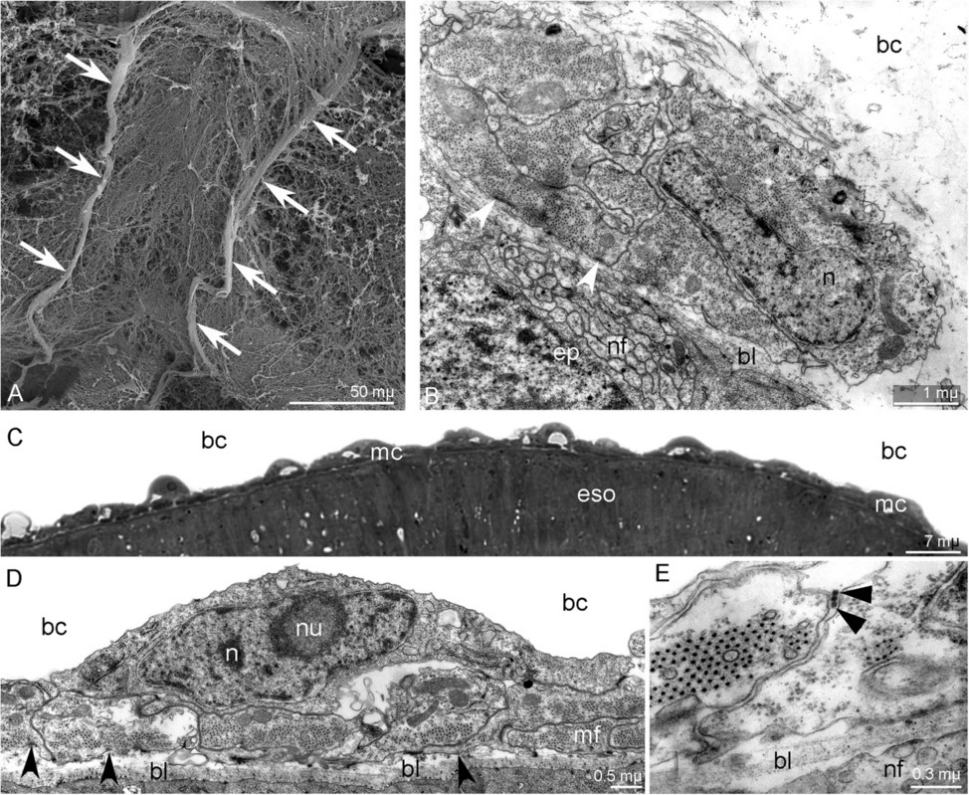
**Organization of the hood elevators and the esophageal musculature in *Phoronopsis harmeri *larvae. A**. Fine morphology of hood elevators (arrows) (SEM). **B**. Cross section of hood elevator; muscle cells contact basal lamina via hemidesmosomes (open arrowheads). **C**. Semi-thin sagittal section of the esophagus (eso) with complete muscular lining (mc). **D**, **E**. Longitudinal fine section of muscular lining of the esophagus. **D**. Muscule cells contact basal lamina via hemidesmosomes (open arrowheads). **E**. Thick collagenous fibers between membranes of muscle cells are indicated by arrowheads (closed arrowheads). Abbreviations: bc – blastocoel; bl – basal lamina; ep – epidermis; mf – myofilaments; nf – nerve fibers.

The hood elevators are paired muscles, which extend between the apical plate and collar ring muscle and pass along the dorsal sides of the preoral lobe and collar (Figures 4C and 6A). Each elevator consists of 4–6 cells, which have a typical fine structure and which connect to the basal lamina via hemidesmosomes (Figure 6B).

The esophageal musculature is composed of numerous muscle cells, which pass around the esophagus and form a continuous layer (Figure 6C). The muscle cells form numerous basal processes (Figure 6D); they do not bear desmosomes, and they contact each other via thick electron-dense collagenous fibers that pass around the esophagus (Figure 6E).

**Figure 7:**
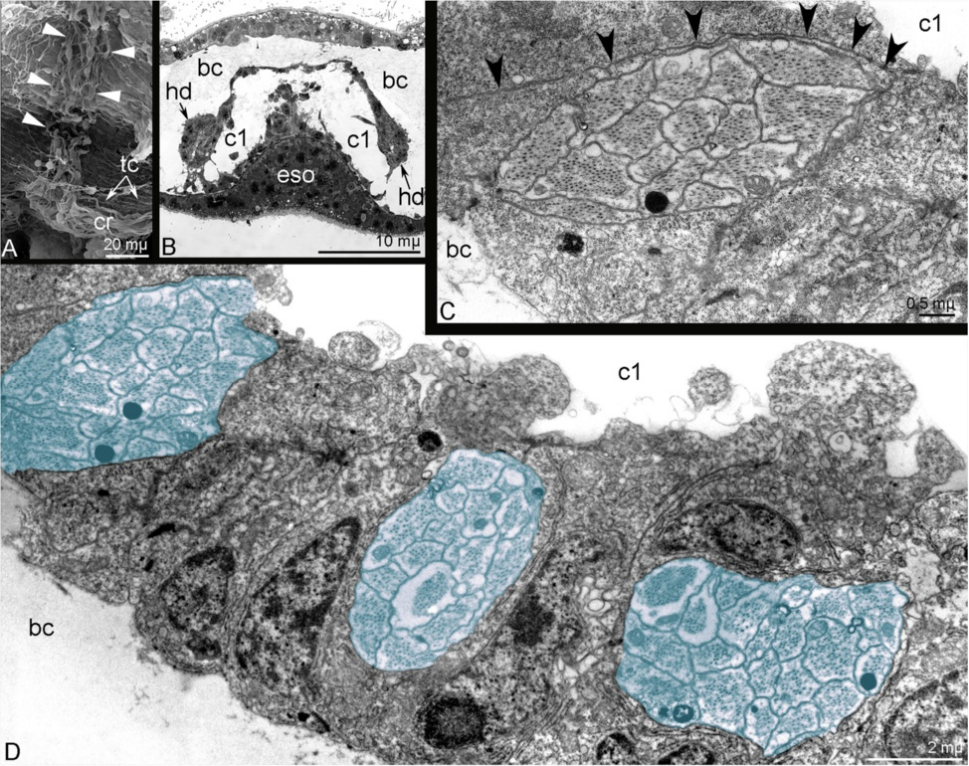
**Hood depressors of *Phoronopsis harmeri *larva. A**. Fine morphology of hood depressor (closed arrowheads), which passes along the wall of the collar to the circular ring muscle (cr). **B**. Semi-thin cross section of the central part of the preoral lobe and preoral coelom (c1) with upper portion of hood depressors (hd). **C**, **D**. Cross sections of the preoral lobe. **C**. Singular branch of the hood depressor is surrounded by thin projections of coelothelial cell (open arrowheads). **D**. Hood depressor muscle bunches (blue) are integrated between the coelothelial cells of preoral coelom (c1) lining. Abbreviations: bc – blastocoel; tc – transverse muscles of the collar.

The hood depressors extend between the apical plate and the collar ring muscle (Figures 4E,G,H). They pass along the ventro-lateral sides of the collar region and closely contact its walls (Figure 7A). At the top of the preoral lobe, the hood depressors are integrated into the lateral walls of the preoral coelom (Figure 7B). Here, each hood depressors divides into several thinner bundles, which penetrate between the cells of the preoral coelomic lining (Figures 4E and 7D). Muscular bundles are surrounded by thin, long processes, which belong to the cells of the coelomic lining (Figure 7C). Each hood depressors bifurcates near the collar ring muscle, and the two branches contact the collar ring muscle (Figures 4B,C).

**Figure 8:**
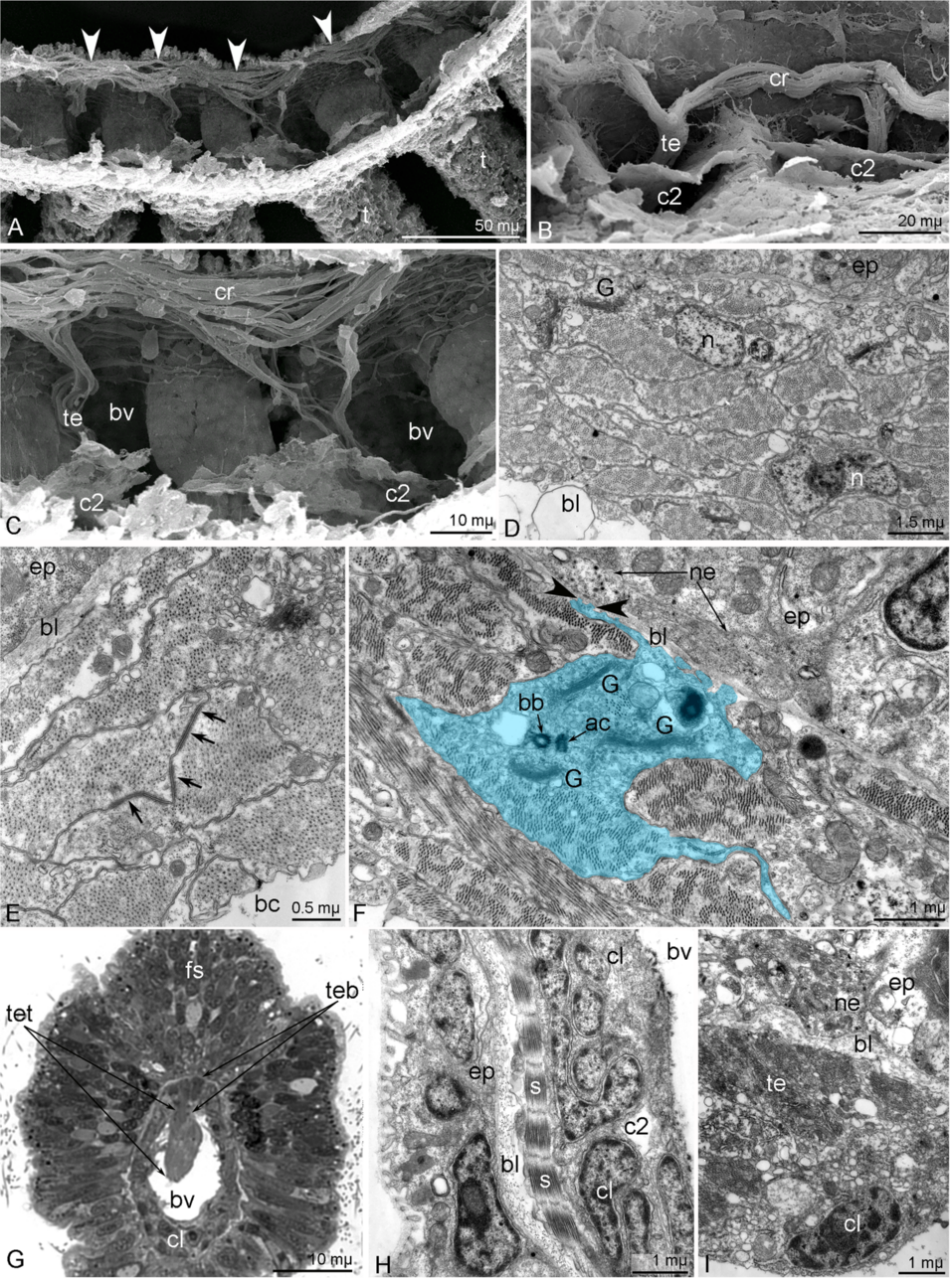
**Fine morphology and ultrastructure of the collar ring muscle and tentacle elevators of *Phoronopsis harmeri *larvae. A** – **C**. Fine morphology of the collar ring muscle and tentacle elevators (SEM). **A**. Overview of the collar ring muscle (white arrowheads). **B**. Part of the collar ring muscle (cr) and two tentacle elevators (te) in a competent larva. **C**. Disorderly net of muscle cells of the collar ring muscle in an advanced larva. **D** – **F**. Ultrastructure of the collar ring muscle (TEM). **D**. Overview of muscle cells with nueclei (n). **E**. Desmosomes (arrows) between muscle cells. **F**. Organization of one of the muscle cells (light blue), which has a cilium, basal body (bb), accessory centriole (ac), several Golgy complexes (**G**), and long processes that spread on the basal lamina (bl) and form bulbs (black arrowheads) opposite the nerve fibers (ne). **G**. Semi-thin cross section of the tentacle in a competent larva shows tentacular blood vessel (bv), coelomic lining (cl), frontal side epidermis (fs), the base of the tentacle elevator (teb), and the terminal bulge of the tentacle elevator (tet). **H**. Part of longitudinal section of the tentacle; a muscle cell with sarcomeres (s) is evident. **I**. muscle cells of the tentacle elevator (te), which is squeezed between the basal lamina (bl) of epidermis (ep) and the cells of the coelomic lining (cl) of the tentacular coelom.

The main element of the larval muscular system is the collar ring muscle (Figures 4D,G). The collar ring muscle extends above the tentacle base in the blastocoel of the collar and is shaped like a horseshoe because it is interrupted on the dorsal side (Figure 8A). The cells that compose the collar ring muscle occupy a large volume and are organized as a wide net in the advanced larva (Figure 8C) but form a thick muscle in which all cells are packed together in the competent larva (Figure 8B). In general, the collar ring muscle consists of more than 40 monociliate cells, which are connected to each other via desmosomes and which contact the basal lamina of epidermis via hemidesmosomes (Figure 8D, E). The muscle cells form numerous long projections. Some of these projections spread on the basal lamina and form small bulbs, which connect to the basal lamina. On the opposite side of the basal lamina, synaptic vesicles are released from the nerve projection (Figure 8F). The center of the muscle cell lacks myofibrils but contains several Golgi complexes and a nucleus, which contains the nucleolus. Mitochondria are scattered in the cell cytoplasm. Each muscle cell has one cilium, which is situated in a deep apical invagination. A basal body and an additional centriole are located in the cytoplasm. Myofibrils form a regular structure with Z-lines and sarcomeres (Figure 8H). All of these characteristics are typical for most muscles of the actinotroch except for the telotroch flexors, the telotroch constrictor, the trunk musculature, and the musculature of the metasomal sack.

The collar ring muscle gives rise to the tentacle elevators, which begin as two branches at the base and continue as thick muscular bands along the frontal (upper) side of each tentacle (Figure 8C). Each tentacle elevator consists of two parts: the base and the terminal bulge. The muscle cells that form the base of the tentacle elevator are squeezed between the epidermis and the cells of the tentacular coelomic lining (Figure 8I). The terminal bulge of the tentacle elevator extends into the tentacular blood vessel (Figure 8G). Cross sections reveal that the base of the tentacle elevator is formed by 4–5 muscle cells and that the terminal bulge is formed by and 7–8 cells (Figure 8G). All of the muscle cells are in close contact but desmosomes between them were not found (Figure 6I). In the advanced larva, numerous muscle fibers occur along the abfrontal (lower) and lateral sides of the tentacle (Figures 4C,D). These are tentacle depressors, which are formed by cells of the coelomic lining of the tentacular coelom. These muscles are very weak, but they can be recognized with phalloidin staining (Figure 4F).

**Figure 9:**
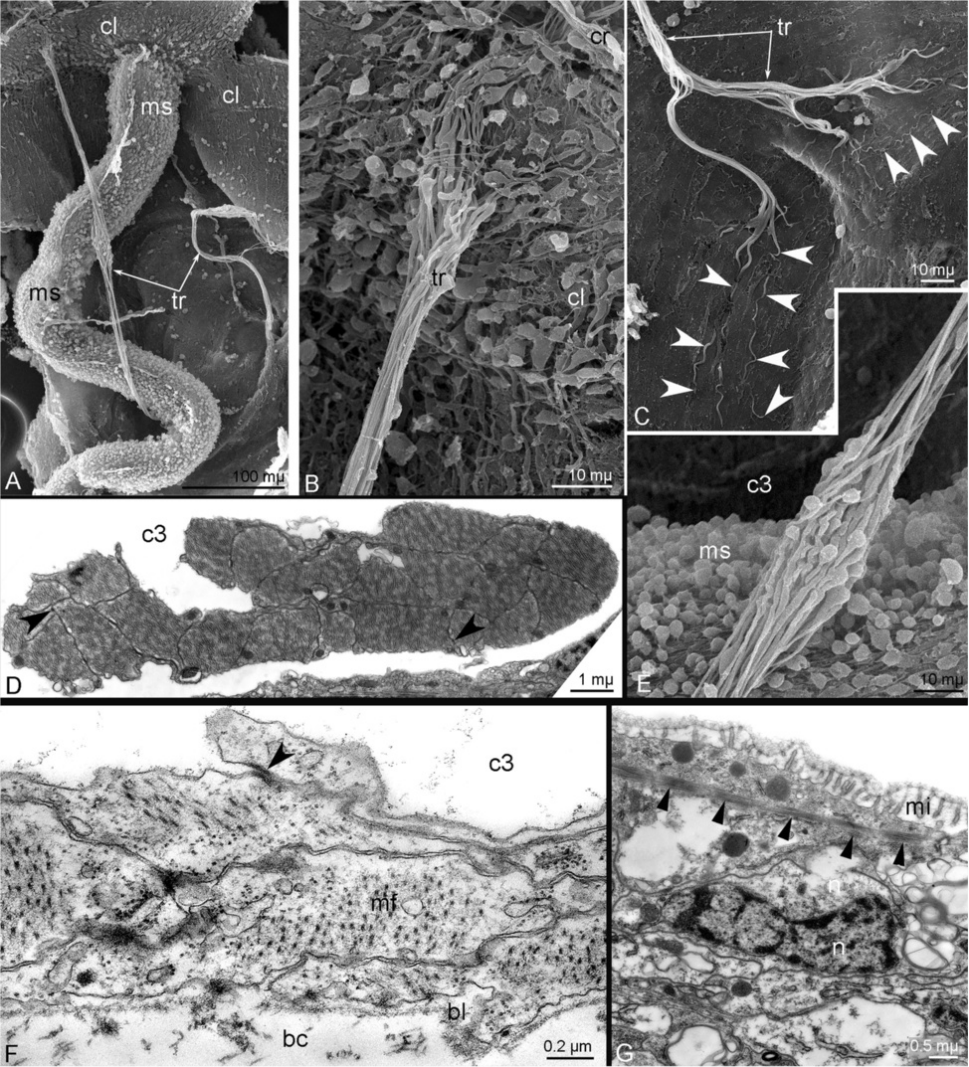
**Organization of trunk retractors, telotroch flexor, and telotroch constrictor in *Phoronopsis harmeri *larvae. A**. Overview of two trunk retractors (tr) in the trunk coelom (SEM). **B**. Upper portion of the trunk retractor contacts the coelomic lining (cl) of the trunk coelom and collar ring muscle (cr) (SEM). **C**. Lower (posterior) portion of trunk retractor forms numerous long processes (white arrowheads) (SEM). **D**. Fine cross section of trunk retractor (TEM). Muscle cells connect to each other via desmosomes (black arrowheads). **E**. Middle portion of the trunk retractor contains nuclei of muscle cells. **F**. Cross section of larval trunk. Cells of the trunk coelom (c3) somatopleura form telotroch flexors and connect to each other via desmosomes (black arrowhead). **G**. Epidermal cells near the telotroch contain cross-striated-like myofilaments (closed arrowheads) in apical cytoplasm (TEM). Abbreviations: bc – blastocoel; bl – basal lamina; c3 – trunk coelom; mi – microvilli; ms – metasomal sack; n – nucleus.

The trunk retractors are paired muscles that cross the trunk coelom from the telotroch to the collar (Figure 9A). Each trunk retractor is composed of more than 20 muscle cells, which form long processes, are connected to each other via desmosomes, and have a cross-striated organization (Figure 9D). The center of each muscle cell is located in the center of the retractor and contains a nucleus (Figure 9E). The upper end of the trunk retractor contacts the collar ring muscle via the coelomic lining of the trunk coelom (Figure 9B). The posterior end of each trunk retractor branches and forms several muscle bundles (Figures 4B and 9C). These bundles consist of several projections of muscle cells, which are very long and which spread along the trunk surface (Figure 9C).

The telotroch flexors (Figures 4H) are formed by the cell of the metacoel lining. These muscles are composed of long projections of muscle cells; the projections overlay each other, are connected via desmosomes, and contact the basal lamina via hemidesmosomes (Figure 9F). The myofibrils are organized as they are in smooth musculature.

**Figure 10:**
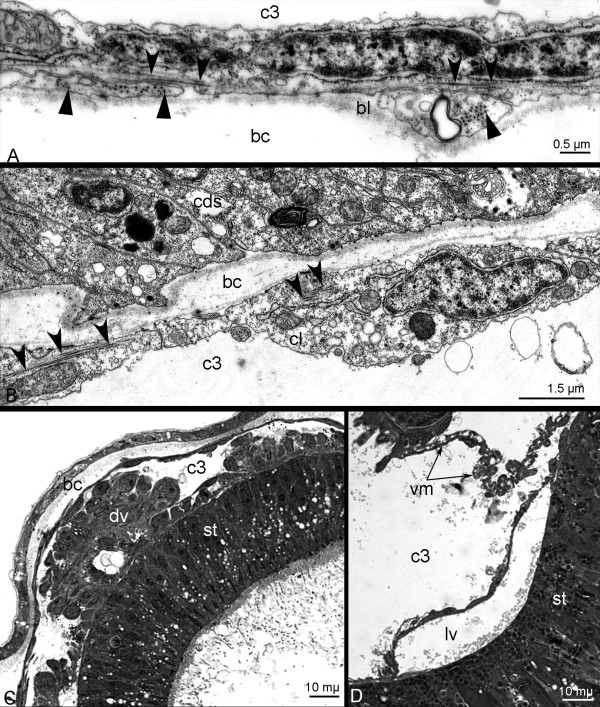
**Metacoel lining and blood vessels in *Phoronopsis harmeri *larvae. A**-**B**: Longitudinal fine sections (TEM); longitudinal myofilaments are indicated by concave arrowheads; circular myofilaments are marked by straight arrowheads. **C**-**D**: Semi thin cross sections. **A**. Lining of the body wall. **B**. Lining of the digestive tract. **C**. The dorsal blood vessel (dv). **D**. The lateral blood vessel (lv). Abbreviations: bc – blastocoel; bl – basal lamina; cds – cells of the digestive system; c3 – trunk coelom; cl – cells of coelomic lining; st – stomach; vm – ventral mesentery.

In other parts of the trunk coelom, the lining of body wall consists of two layers (Figure 10A). An external layer forms the circular musculature and contains sporadic transverse muscular cells. An internal layer consists of flat cells with singular longitudinal myofibrils in the basal cytoplasm. The lining of digestive tract is composed of coelothelial cells, which usually contain a few myofilaments and have a very weak muscular organization (Figure 10B). Strong muscle filaments occur in coelothelial cells, which form the wall of the larval blood vessels (for details see [17,18]). The lining, which forms the wall of the dorsal blood vessel, has the most developed musculature in comparison to musculature of metacoel lining in other parts of the digestive tract (Figure 10C). This vessel can be recognized even in live larvae because of its thick wall, large lumen, and regular contractions. In semithin sections, the dorsal blood vessel has a thick wall that consists of external tall coelothelial cells and an internal lining (Figure 10C). Flat cells and their numerous long, basal processes of lining of the digestive tract form the lateral blood vessels of larva (Figure 10D).

The telotroch constrictor is formed by epidermal cells, which are located above the telotroch. The myofibers are grouped in parietal bundles and extend into the apical cytoplasm of these cells (Figure 9G). The myofibrils exhibit a cross-striated-like organization (Figure 9G).

### Metamorphosis

**Figure 11:**
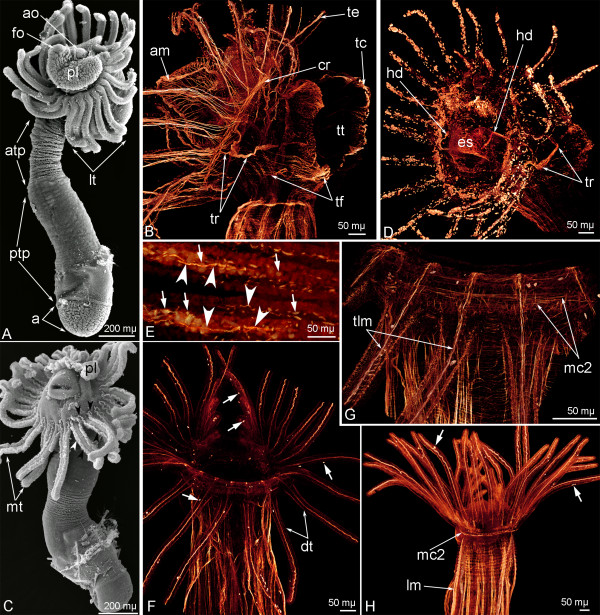
**Metamorphosis of *Phoronopsis harmeri*.** In all images, the apical is to the top. **A**. First stage of the metamorphosis (3 minutes after metamorphosis has begun) – animal with completely everted metasomal sack, which forms the juvenile body, is divided into the anterior trunk part (atp), the posterior trunk part (ptp), and the ampulla (a) (SEM). **B**. Head region of animal at the first step of metamorphosis, stained with phalloidin. 3D-reconstruction of muscular system; lateral view. **C**. Second step of the metamorphosis (10 minutes after metamorphosis has begun) – animal with engulfed preoral lobe (pl) (SEM). **D**. The same stage, 3D-reconstruction of musculature of animal head region stained with phalloidin; ventro-lateral view. **E**. Tentacles with degenerated (arrows) and complete (arrowheads) muscles. **F**. Oral view of newly formed juvenile 20 minutes after metamorphosis has begun. 3D-reconstruction of musculature of head region. Degenerated muscles are shown by arrows. **G**. The same stage; higher magnification of tentacles and lophophore with newly formed tentacle longitudinal muscles (tlm) and circular muscle of the tentacular coelom (mc2). **H**. Oral view of head region of 4-day-old juvenile. Degenerated muscles are shown by arrows. Abbreviations: am – annular muscle of the preoral lobe; ao – apical organ; cr – collar ring muscle; dt – definitive tentacles; es – esophageal musculature; fo – frontal organ; hd – hood depressor; lm – longitudinal muscles of the trunk; mt – metamorphic tentacles; tc – telotroch constructor; te – tentacle elevator; tf – telotroch flexor; tr – trunk retractor; tt – telotroch.

**Figure 12:**
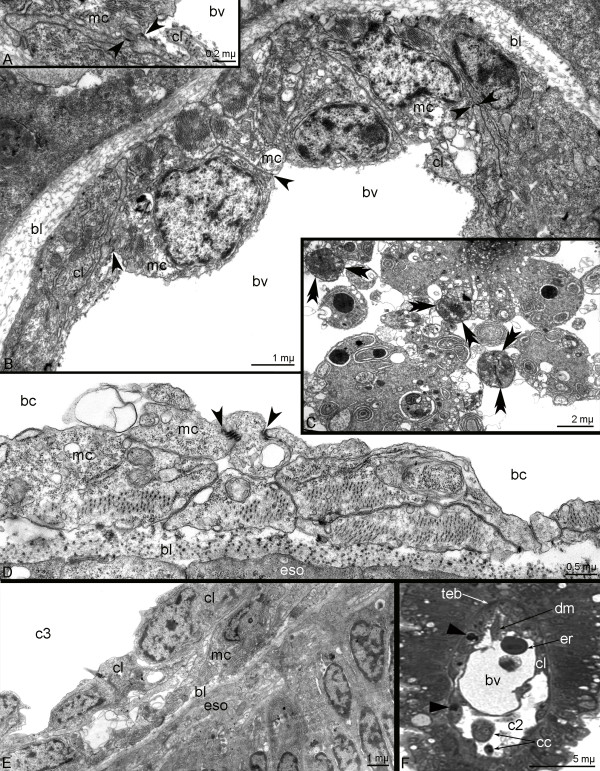
**Organization of musculature in 4-day-old (A-C, E-F) and metamorphic (D) *Phoronopsis harmeri*. A**. Borders with desmosome-like contacts (open arrowheads) between muscle cell (mc) of the tentacle elevator base and cell of the tentacular coelom lining (cl). **B**. Cross section of the tentacles with muscle cells of the tentacle elevator base, cells of the coelomic lining, and blood vessel (bv). Desmosomes between two muscle cells and between the muscle and coelothelial cells are indicated by opened arrowheads. **C**. Coelomocites include parts of degenerated muscular cells (double arrowheads). **D**. Longitudinal section of esophageal muscular lining with desmosomes (arrowheads) between cells. **E**. Coelomic lining (cl) of the trunk coelom (tc) and esophageal musculature (mc); longitudinal section of the esophagus (eso). **F**. Semi-thin cross section of the tentacle with blood vessel and degenerated muscles (dm) and erythrocyte (er) inside, base of the tentacle elevator (teb), coelomic cavity (c2) with coelomocites (cc), cells of the coelomic lining contain huge phagosomes (closed arrowheads).

Phoronid metamorphosis begins with a great contraction of the hood depressors and trunk retractors (Additional file 1). At this moment, the larval body decreases in length by two to three times. This contraction generates high pressure in the trunk coelom and causes the eversion of the metasomal sack, which takes about 3 minutes (Additional file 1). The telotroch is pulled into and kept within the larval body by the telotroch constrictor. During the first step of the metamorphosis, the dorsal side of larval body decreases in length, whereas the ventral body side (metasomal sack is an extension of larval ventral body side) greatly increases in length and gives rise to the adult body (Figure 11A). In the first metamorphic stage, all main larval muscles can be recognized with phalloidin (Figure 11B). Then, the muscular system of the preoral lobe degenerates and is engulfed together with the preoral lobe (Figure 11C). Larval tentacles also undergo changes: the epidermis of the postoral ciliated band, which contains specialized latero-frontal cells, peels off and then is ingested (Figure 11C). The larval tentacles, which lack the postoral ciliated band, directly become the tentacles of juvenile. The destruction of the larval muscular system begins 10 minutes after metamorphosis has begun. The collar ring muscle and tentacle elevators turn into huge globular conglomerates, which fill the anterior volume of the juvenile (Figure 11D). Some muscles cells that form the tentacle elevators retain their integrity and can be recognized by both confocal and transmission electron microscopy (Figures 11E and 12B). Transmission electron microscopy reveals the integration of some elements of the larval muscular system into the juvenile muscular system. This integration involves tentacle elevators and the esophageal musculature. In larvae, we could not detect desmosomes in these muscles even though we studied fine sections of different parts of the tentacles and the esophagus. At the same time, in newly formed juvenile, after metamorphosis has proceeded for 10 minutes, desmosome-like contacts appear between the muscle cells that form the base of the tentacle elevator and the cells lining the tentacle coelom (Figures 12A, B). Cells of the terminal bulge of the tentacle elevator are destroyed and their remnants lay in the tentacular blood vessel, which is directly derived from the tentacular blood vessel of the larva. The integration via desmosomes is evident in the muscle cells forming the esophageal musculature. At first, the desmosome-like contacts appear between the muscle cells of the esophageal musculature (Figure 12D), and then the esophageal musculature fuses with the lining of the trunk coelom and forms the muscular lining of the adult esophagus (Figure 12E). This integration, which appears to result from desmosome formation, allows the muscular system to rebuild within 10 minutes after the start of degeneration (Figure 11F). Destroyed muscular cells are consumed by numerous coelomocytes, which can be observed with TEM (Figure 12C), and large digestive vacuoles can be recognized in the cells of the coelomic lining (Figure 12F). The definitive musculature of the tentacles mostly originates from cells of the tentacular coelom lining. The volume of the tentacular coelom increases, whereas the volume of the collar blastocoel decreases, and the blastocoel gives rise to lophophoral ring vessels. The base of the tentacular coelom gives rise to the mesosomal musculature (Figure 11G). The muscular system of the anterior part of the juvenile appears quickly and becomes stronger with time (Figure 11H), although some globular conglomerates are evident in 4-day-old animals (Figure 11H). Interestingly, the hood depressors and trunk retractors, which are very important for body plan formation (they constantly maintain tension between the anterior and posterior ends of the larval body), can be recognized when other muscles are destroyed (Figure 11D). At later stages of metamorphosis, when the apical plate of the larva is engulfed and the preoral coelom changes greatly, the upper portions of the hood depressors degenerate, and the hood depressors clutch at the esophageal musculature, whereas their posterior remnants fuse to the trunk retractors (Figure 11D).

## Discussion

### Myogenesis and organization of muscles in advanced larva

In all phoronid species studied to date, the first muscle cells arise from cells that immigrated from the anterior wall of the archenteron of the embryo at the mid-gastrula stage ([10,13,19,20], therein). Muscle cells arising from this anterior source give rise to the muscles of the preoral lobe, the collar region, and the tentacles ([10], therein). They also give rise to the coelomic lining of the preoral and tentacular coeloms of *P. harmeri*. The preoral coelom originates simultaneously with musculature of the preoral lobe, but cells of the protocoel lining do not have myofibers [18] and can not be traced with phalloidin labelling. The tentacular coelom originates in young *P. harmeri* larva, which has two pairs of tentacles. The trunk coelom and musculature including the telotroch flexors and trunk retractors develop in *P. harmeri* larva later and arise from the pouch in the posterior part of the archenteron, which forms during embryogenesis in several phoronid species [19,20]. The anterior and posterior sources of of the mesoderm are located on the border between the ectoderm and endoderm, which confirms the view that these sites have a main role in mesoderm formation [10].

In phoronid development, the muscles first arise in the preoral lobe ([13,21], herein). According to TEM data [21], *Phoronis muelleri* larva also has muscular cells in the preoral lobe. These cells apparently show an apicobasal polarity, but are never connected each other by apical adherens junctions and do not form true epithelium [21]. The arrangement of circular and radial muscles in the preoral lobe is the same in all phoronid larvae studied to date ([10,12-14,21], herein). The musculature of the preoral lobe allows larvae to create movements that generate suction in the absence of hood elevators. These movements are very important for the capture of food particles and have been described in advanced larvae of different phoronid species [22,23]. The use of musculature for the capture of food particles or for the transport of food particles to the mouth is unknown for other planktotrophic larvae, which usually capture food particles via a ciliary mechanism.

Simultaneous with the development of the preoral lobe musculature, the esophageal musculature arises. The esophageal musculature has a very complex organization and the muscular activity of the esophagus is under nerve control, which is mediated by the net of nerve fibres and some perikarya around the esophagus [24,25]. In planktotrophic larvae of marine invertebrates, the esophageal musculature develops first, becomes strong with age, and in advanced larvae, the esophagus has its own innervations via a net of perikarya and neurites that develops around the esophagus [26-32].

Previous reports have mentioned the formation of transversal muscles of the collar in all young phoronid larvae that have been studied ([10,13], and ref. therein). The collar musculature consists of numerous paired muscles, which arrange repetitively along the oral field of the larva [10]. The thick musculature of the oral field is innervated by numerous neurites and perikarya, which are scattered in the oral field epidermis [25]. The abundance of the muscular and the nervous elements in the oral field is likely related to the functional significance of this body part, which is involved in the transport of food particles from the tentacles to the mouth [22,23].

The next step of myogenesis is the formation of the collar ring muscle. It’s appearance connects with formation of primordial of the tentacles, which develop as outgrowths of the oral field. The collar ring muscle is present in some phoronid larvae and absent in other. Thus, in larvae of *P. harmeri* and *P. pallida*, the collar ring muscle appears in young larvae and remains the most prominent muscle during larval development ([12,13], herein). In competent actinotrocha C, actinotrocha D, and *Phoronis architecta* larvae, the collar ring muscle is not evident [14]. Interestingly, in the case of *P. ijimai*, the collar ring muscle is absent in young larvae until stage with six tentacle [10,33], but is present in competent larva [own data]. Moreover, in young *P. ijimai* larvae, tentacle elevators are also absent. At the same time, hood depressors in these larvae form long posterior branches that penetrate into the tentacles and probably fill the functions of tentacle elevators [10,33].

The collar ring muscle functions as an anchor for the hood depressors and trunk retractors and gives rise to the tentacle elevators [8]. Hood depressors were described in *P. harmeri* larvae only [8, herein], although they can be observed in actinotrocha D [14] and in larvae of *P. pallida*[13] and *P. ijimai*[10,34].

The last event in myogenesis is the formation of the trunk musculature. The telotroch flexors and trunk retractors originate simultaneously. Although the telotroch flexors have been mentioned in all phoronid larvae studied to date (herein, [10,11]), in one report [11], these muscles are referred to as trunk retractors and in another paper [10], these muscles are not evident at all. In general, the telotroch flexors are well developed in small phoronid larvae (like the larvae of *P. ijimai* and *P. pallida*), which swim actively by using their strong telotroch, in contrast to large phoronid larvae (like the larvae of *P. harmeri*), which float in the water and usually do not swim quickly [8].

Using light microscopy, Zimmer was the first to describe the trunk retractors in *P. harmeri* larvae [8]. Our results, which were obtained with cytochemistry and TEM, confirm and amplify Zimmer’s data. The trunk retractors are very important for the formation of new body plan in the juvenile (see below). According to literature [12], larvae of *P. pallida* lack distinct trunk retractors and other muscles provide the formation of new body plan.

The telotroch constrictor is described here for the first time. This muscle has an unusual organization and is formed by epidermal cells with apical myofilaments. Phoronid larvae have myoepithelial cells in the epidermis of different body parts. Thus, myoepithelial cells have been found in the epidermis of the esophagus [24], the apical organ, the preoral ciliated band, and the oral field [5]. Myoepithelial cells also occur in the epidermis of larvae of other invertebrates; for example, in the apical organ [34] and in the epidermis of prototrochs [35] of pelagic larvae of some entoprocts.

Our data shows that there is a difference in development and organization of the muscular system in larvae of different phoronid species. This difference was assumed before [8] and, probably, corresponds to the type of larva [11].

### Metamorphosis

Although phoronid metamorphosis has been described several times based on light microscopy and histological methods [36-38], the fate of larval musculature has been traced only once and not in detail [12]. According to the latter study, all larval muscles undergo cell death and degenerate [12]. Our results show that the *P. harmeri* larva contains some muscles that do not undergo cell death and that are inherited by the juvenile (Table 1). This difference in the fate of larval musculature probably reflects the presence of two pathways of phoronid metamorphic remodeling.

**Table 1:** The presence of muscles in larvae of two phoronid species and the fate of these muscles during metamorphosis

**Muscle**	**Found in larva / Fate in metamorphosis**	
	***Phoronopsis harmeri *(this paper)**	***Phoronis pallida *[**[10,11]**]**
1. Annular muscle of the hood	+ but lost in the first minutes	+ and then lost
2. Radial and circular muscles of the hood	+ but lost in the first minutes	+ and then lost
3. Hood depressors	+, retained for 15 minutes, and then lost	+ (referred to as “hood elevators”) and then lost
4. Hood elevators	+ and then lost	+ (are not shown) and then lost
5. Esophageal musculature	+ and then incorporated in the juvenile body	+ / ?
6. Transverse muscles of collar	+ and then lost	+ and then lost
7. Collar ring muscle	+ but lost in 10 minutes	+ and then lost
8. Tentacle elevators	+ and then incorporated in the juvenile body	+ and then lost
9. Tentacle depressors	+ and then lost	+ and then lost
10. Trunk body musculature including muscles of the blood vessels	+ and then incorporated in the juvenile body	+ (dorsal blood vessel of larva referred to as “dorsal retractor”) and then lost
11. Trunk retractors	+ , retained for 15 minutes, and then lost	___
12. Telotroch flexors	+ and then lost	+ and then lost
13. Telotroch constrictor	+, retained for 9 days, and then lost	___
14. Metasomal sack musculature	+ and then gives rise to the musculature of the juvenile trunk body wall	? / ?

"+" stands for presence of muscles in larva;

"-" stands for absence of muscles in larva;

"?" is used if information absence.

In phoronids, the formation of a new body plan connects with enormous outgrowth of the ventral side and the shortening of the dorsal side of the larva. In *P. harmeri*, there are some muscles, which are definitely important for formation of a new body plan: the hood depressors, the trunk retractors, and the telotroch constrictor. All these muscles can be observed at the later stages of metamorphosis when most of other muscles are destroyed. Muscles of the first two groups are the most important for the formation of the juvenile body plan because they bring together the anterior and posterior ends of the larva and thereby reduce larval body length. The work of both the anterior (hood) and posterior (trunk) muscles are important. The hood depressors pull on the esophagus so that it assumes its definitive straight position. With contraction of the posterior (trunk) retractors, the dorsal side of the larva gradually shortens.

Larvae of *P. pallida* lack distinct trunk retractors have two retractor muscles that insert at the midpoint of the gut and are involved in movements of the gut during metamorphosis [12]. The “dorsal retractor”, which was found in *P. pallida* larvae, is also involved into metamorphosis and helps to bend the larva at its dorsal midpoint [12]. In *P. harmeri* we did not find any special retractors of the metasomal sack. The metasomal sack and the gut are joined with the ventral mesentery, which gives rise to the oral–anal mesentery of the juvenile [18,39]. Because of the connection, the digestive tract pulls into the metasomal sack when it everts during the first minute of metamorphosis. The “dorsal retractor” evidently corresponds to dorsal blood vessel, which has mostly circular muscles [18] and can not be involved into bending of the larva.

The telotroch constrictor is important for reorganization of the posterior part of the larva. After the trunk retractors contract, the telotroch is pulled into the larval body, and the contraction of telotroch constrictor locks the telotroch within (Additional file 1). The telotroch remains in this position for 9 days, during which time the epidermis of the telotroch disintegrates into cellular debris [21].

In *P. harmeri*, the esophageal musculature is retained during metamorphosis and provides swallowing movements when some parts of larval body are consumed by the juvenile.

According to literature [12], the degeneration of tentacle depressors and elevator means that the newly formed juvenile cannot perform muscular movement of its tentacles (tentacular flicks). In *P. harmeri*, metamorphosis occurs in another way and the basal parts of each tentacle elevator remain during metamorphosis and integrate into the mesocoel lining. Because of this integration, *P. harmeri* retains the ability to perform muscular movement of its tentacles at all stages of metamorphosis. The integration of cross-striated larval muscles into the juvenile musculature is indicated by some cytological evidence. In juvenile and adult phoronids, in each tentacle, a strong longitudinal cross-striated muscle passes along the frontal side [40-42]. The location and fine structure of this muscle corresponds to the position and fine organization of the larval tentacle elevator.

On the other hand, remodeling of the tentacle muscular system in phoronids seems strange because larval and definitive tentacles have the same function and their muscles make similar movements. Thus, the contraction of the larval tentacle elevator lifts the larval tentacle. In adults, the contraction of the frontal longitudinal muscle of the tentacle bends the tentacle and produces a “flick”-like movement, which is important for the capture of food particles [23]. The remodeling of the tentacle muscular system in phoronids probably corresponds with the special respiratory function of the adult tentacle [12].

The lining of the larval digestive tract is inherited by the juvenile and retains its muscular organization in the adult. In adult *P. harmeri*, the lining of digestive tract consists of myoepithelial cells with longitudinal myofibres; circular muscles are absent, and basal projections of the stomach epithelium, which contain circular myofibers, function as a circular musculature [43]. The juvenile inherits blood vessels, which are built in the larva. The dorsal blood vessel of the larva gives rise to the median blood vessel of the adult. The “dorsal retractor”, which is mentioned in Santagata’s article [12], might correspond to the dorsal blood vessel, which has thick muscular walls and can be observed with phalloidin staining.

### Organization of the muscular system in phoronid larvae and other ciliated larvae of marine invertebrates

The organization of the larval muscular system mostly depends on larval life style; therefore, it is usually very difficult to establish the homology between muscular elements in larvae of different animals [30]. In ciliated larvae, the most important organs generally have their own musculature, and these muscle elements are innervated by prominent nerve tracts. The most prominent organs of marine pelagic ciliated larvae are ciliated bands. Ciliated bands contain sensory cells that pass excitation to the intraepidermal neurites, which then stimulate the muscles. Thus, in *P. harmeri* larvae, all ciliated bands (preoral, postoral, and telotroch) are controlled by thick muscles (annular hood muscle, circular collar muscle, and telotroch constrictor muscle), which are innervated by well-developed nerve tracks (marginal nerve of the preoral lobe, tentacular neurite bundle, and telotroch nerve rings) [25]. This co-localization is most evident in ciliated bands, the esophagus, and the apical organ. The same co-localization (overlapping) of muscles and nerve tracts is known in marine larvae of nemerteans [44], bryozoans [45-47], annelids [29,48], echinoderms [49], hemichordates [50], brachiopods [51], and many other invertebrates. Pelagic larvae of marine invertebrates usually have a well-developed apical organ, which can be withdrawn into the larval body via contraction of the apical muscle. This muscle, which extends between apical plate and body wall or wall of the digestive tract, occurs in tornaria ([32]: Figure 4), bryozoan larvae [46,52], entoproct larvae [53], and nemertean larvae [44]. If the larva is threatened, the contraction of this muscle pulls the apical organ into the larval body. Phoronid larvae also have special muscles—the hood depressors—that pull the apical organ into the larva and protect it.

Concerning the fine structure of larval muscle cells, the difference in the ultrastructure of muscle cells in lophotrochozoan and deuterostomian larvae is important. Usually, lophotrochozoan larvae have cross-striated muscles, whereas deuterostomian larvae have only smooth muscles [54-56]. Although some data indicate that some larval and juvenile musculature appears to be obliquely striated with discontinuous z-bands [12], we did not find this type of muscle organization in *P. harmeri* larvae. According to our results, phoronid larvae have both cross-striated and smooth muscles. Those muscles that originate from an anterior mesoderm are usually cross-striated, whereas those muscles that originate from a posterior mesoderm are usually smooth. These data do not definitely indicate a protostomian affiliation for phoronids but probably support that phoronids combine some deuterostome-like and protostome-like features [25].

### Remarks on phylogeny

Although previous researchers have attempted to establish the homology between different muscles in different Bilateria [7], doing so is in most cases very difficult because the organization of the muscular system usually depends on the peculiarities of biology and life style. For example, a comparative analysis of musculature in phoronids and brachiopods was unsuccessful [30]. In any case, comprehensive information about the development and organization of the muscular system may help in future analysis.

Comparative analysis is complicated by the remodeling of the muscular system that occurs during bilaterian metamorphosis. Among phoronids, two main patterns of muscular system remodeling are evident during metamorphosis: complete and incomplete destruction of larval muscular elements. In *Phoronopsis harmeri*, some muscular elements of the larva integrate into the juvenile musculature, whereas in *Phoronis pallida*, the larval musculature is completely destroyed as the larva develops into the juvenile. This difference may reflect the phylogenetic status of the genus *Phoronopsis*, whose members exhibit more plesiomorphic conditions than those in the genus *Phoronis*[57]. The plesiomorphic nature of *Phoronopsis* together with its retention of larval tentacular musculature and its direct transformation of larval tentacles into juvenile tentacles suggests that the presence of tentacles is a primary phoronid characteristic, i.e., the phoronid ancestor had tentacles at larval and adult stages. In that case, the absence of tentacles in *P. ovalis* larvae can be regarded as a derived condition. Moreover, because recent research indicates that phoronids are nested with brachipods [3] and because some brachiopods have planktotrophic larvae with tentacles, we suggest that common brachiozoan ancestor had tentacles that were used to feed. Therefore, the presence of planktotrochic larvae may be regarded as primary condition of brachiopods. This statement has some support from paleontological data [58], although the primacy of planktotrophy vs. lecithotrophy in bilaterian phylogeny generally remains unclear [59].

Interestingly, brachiopods also exhibit two types of metamorphosis: in the lecitotrophic larvae of some rhynchonelliform (articulate) brachiopods [30] and *Novocrania anomala*[60], the larval musculature is destroyed and juvenile muscles appear *de novo*. The planktotrophic larvae of *Glottidia* and *Lingula*, in contrast, retain the larval musculature during metamorphosis [51]. Both phoronid larvae and brachiopod planktotrophic larvae undergo some changes in their lophophoral musculature, which correlate with a switch from swimming and feeding in the plankton to burrowing and feeding in the benthos. Despite these changes, both phoronids and brachiopods exhibit specific peculiarities in filter-feeding mechanisms, which can be observed in both larval and adult stages [61]. Strathmann [61] described the combination of some protostome-like and deuterostome-like features in the organization of ciliated bands and filter-feeding mechanisms in phoronids and brachiopods.

The combination of protostome-like and deuterostome-like features in phoronids described previously [5,25,39] and in this report probably reflects the basal position of brachiopods and phoronids within the Lophotrochozoa, as discussed by Peterson and Eernisse [62]. The view that brachiopods and phoronids occupy a basal position within the Lophotrochozoa contradicts recent molecular data [1,2] but must be examined comprehensively with different modern methods.

## Conclusion

This report is the first detailed report about the development and organization of the muscular system in phoronids from fertilization through metamorphosis to juvenile. The first muscle cells originate from the anterior mesoderm and occupy the anterior portion of the embryo. The muscle of the preoral lobe is the first muscle to appear, and it plays a main role in food capture. Two ventrolateral muscles and transverse repetitive muscles then appear on the ventral side of the collar. The organization of the muscular system in competent phoronid larvae is very complex and at least 14 groups of muscles are evident. During metamorphosis, most larval muscles degenerate and turn into huge globular conglomerates. The hood depressors and trunk retractors remain trough the metamorphosis and play important role in formation of juvenile body plan, because these muscles tie together anterior and posterior parts of larval body. Some larval muscle elements, such as the tentacle elevators and, esophageal musculature, and metacoel lining are inherited by the juvenile. Our results and literature data allow to conclude that among phoronids, two main patterns of muscular system remodeling during metamorphosis: complete and incomplete destruction of larval muscular elements.

The muscular system of phoronid larvae is generally more complex than that of the ciliated larvae of other marine invertebrates. This complexity reflects the long active life of phoronid larvae in the plankton, planktotrophy, and catastrophic metamorphosis, the latter of which is mediated by specialized muscles. Some organs (the apical organ, the ciliated bands, the esophagus) are supported by similar muscle elements in the larvae of different invertebrates.

The fine organization of phoronid muscles combines protostome-like and deuterostome-like features. This combination, which has also been found in the organization of some other systems in phoronids, can be regarded as an important characteristic and one that probably reflects the basal position of phoronids within the Lophotrochozoa.

## Methods

The myogenesis, organization of larval muscular system and its metamorphic remodeling were studied in *Phoronopsis harmeri* Pixel, 1912.

For myogenesis studying, adult *Phoronopsis harmeri* were collected from May to June 2010 in Coos Bay, Oregon, USA, from intertidal sandy sediments. Fertilized eggs, which were extracted from reproductive females by opening the trunk, were kept in glass beakers containing filtered sea water; the temperature of the egg suspension was maintained at 3-14°С by keeping the beaker partially submerged in running sea water on a laboratory bench. Under these conditions, embryos developed normally. Within 15 min of exposure to sea water, two polar bodies were formed and cleavage commenced. Stages of development were monitored with a stereo microscope. Larval cultures had a density of two larvae per 3–4 ml filtered sea water. Larvae were fed mixtures of *Rhodomonas lens* and *Chaetoceros calcitrans* and 75% of the sea water was changed every 2 days. At 4 h intervals (up to the 24-day-old larva), specimens were prepared for future investigations (see below).

For larvae and metamorphosis studies, competent larvae of *P. harmeri* were collected with a planktonic net during November of 2011 in Vostok Bay, Sea of Japan. Larvae were reared at 13 to 14C in an incubator with a 12-h light–dark cycle until metamorphosis. At 2–3 min intervals (up to the newly formed juvenile), specimens were prepared for future investigations (see below).

Live embryos were photographed using a Leica DFC 400 camera mounted on an Olympus BX51 microscope. Competent larvae, metamorphic stages, newly formed juveniles, and 4-day-old juveniles were photographed using a Panasonic DMC-TZ10 digital camera mounted on a binocular light microscope. All these stages were prepared for scanning electron microscopy (SEM), transmission electron microscopy (TEM), cytochemistry, and confocal laserscanning microscopy (CLSM).

For SEM, fixed competent larvae and metamorphic stages of *P. harmeri* that had been dehydrated in ethanol followed by an acetone series were critical point dried and then sputter coated with platinum-palladium alloy. Before coating, some larvae were dissected for study of muscles fine morphology. Specimens were examined with a CamScan S2 scanning electron microscope.

For TEM, early embryos, competent larvae, and metamorphic stages of *P. harmeri* were fixed at 4°C in 2.5% glutaraldehyde in 0.05 M cacodylate buffer containing 21 mg/ml NaCl and then postfixed in 2% osmium tetroxide in the same buffer containing 23 mg/ml NaCl. Postfixation was followed by *en bloc* staining for 2 h in a 1% solution of uranyl acetate in distilled water. Specimens were then dehydrated in ethanol followed by an acetone series and embedded in Spurr resin (Sigma Aldrich). Semi-thin and thin sections were cut with a Reichert Ultracut E ultratome. Semi-thin sections were stained with methylene blue, observed with Zeiss Axioplan2 microscope and photographed with an AxioCam HRm camera. Thin sections were stained with lead citrate and then examined with a JEOL JEM 100B electron microscope.

For cytochemistry, embryos, advanced and competent larvae of *P. harmeri*, newly formed juveniles, and 4-day-old juveniles were narcotised in MgCl2, then fixed for 60 min in a 4 % paraformaldehyde solution on a filtrate of sea water and washed (two times) in phosphatic buffer (pH 7.4) (Fisher Scientific) with Triton X-100 (0.1%) (Fisher Scientific, Pittsburgh, PA, USA) and 0.1% albumine bovine (PBT/BSA) (Sigma-Aldrich, St. Louis, MO, USA) for a total of 20 min. Then, the specimens were washed in PBT/BSA and incubated in a mixture of rhodamine-conjugated phalloidin (1:50) (Fisher Scientific, Pittsburgh, PA, USA) and Hoechst (1:1000) (Fisher Scientific, Pittsburgh, PA, USA) for 1 h at RT in the dark. In the following, they were washed in PBS (three times × 15 min), mounted on a cover glass covered with poly-L-lysine (Sigma-Aldrich, St. Louis, MO, USA), and embedded in Murray Clear or Vectashield (Vector Laboratories Inc., Burlingame, CA, USA). Embryos and early larvae were viewed with an Olympus confocal microscope (OIMB, OR, USA). Competent larvae, metamorphic stages, and newly formed juveniles were viewed with an Olympus confocal microscope (Institute of Developmental Biology, Moscow, Russia). Advanced larva of *P. harmeri* was viewed with a Biorad MRC-600 series confocal microscope (Friday Harbour Labs). Z-projections were generated using the programme Image J version 1.43. Three-dimensional reconstructions were generated using Amira version 5.2.2 software (Bitplane, Zurich, Switzerland).

### Terminology

In this report, we have used the terms suggested by Russel Zimmer (1964), who made the most complete description of muscular system of phoronid larvae using methods of light microscopy. We also used some terms that were suggested by Scott Santagata (2004).

## Competing interests

The authors declare that they have no competing interests.

## Authors’ contributions

ENT designed and coordinated research, performed research, analyzed data, and prepared all figures, and wrote the manuscript. EBT performed staining and confocal research of muscular system in competent larvae, metamorphic animals, and juveniles. Both authors conceived the study, read, and approved the final version of the manuscript.

## Supplementary Material

Additional file 1 **The metamorphosis of a *Phoronopsis harmeri *larva.** This time-lapse movie shows a typical sequence of phoronid metamorphosis, which starts with the great contraction of the hood depressors and trunk retractors and eversion of the metasomal sack. Click here for file
